# Exploring the Horizons of Four-Dimensional Printing Technology in Dentistry

**DOI:** 10.7759/cureus.58572

**Published:** 2024-04-18

**Authors:** Sucharitha Palanisamy

**Affiliations:** 1 Periodontics and Oral Implantology, Sri Ramaswamy Memorial (SRM) Dental College and Hospital, Chennai, IND

**Keywords:** fourth-dimensional printing, three-dimensional printing, additive manufacturing, dental applications, smart materials

## Abstract

In dentistry, the integration of additive manufacturing, particularly 3D printing, has marked significant progress. However, the emergence of 4D printing, which allows materials to change shape dynamically in response to stimuli, opens up new avenues for innovation. This review sheds light on recent advancements and potential applications of 4D printing in dentistry, delving into the fundamental principles and materials involved. It emphasizes the versatility of shape-changing polymers and composites, highlighting their ability to adapt dynamically. Furthermore, the review explores the challenges and opportunities in integrating 4D printing into dental practice, including the customization of dental prosthetics, orthodontic devices, and drug delivery systems and also probing into the potential benefits of utilizing stimuli-responsive materials to improve patient comfort, treatment outcomes, and overall efficiency and the review discusses current limitations and future directions, emphasizing the importance of standardized fabrication techniques, biocompatible materials, and regulatory considerations. Owing to its diverse applications and advantages, 4D printing technology is poised to transform multiple facets of dental practice, thereby fostering the development of healthcare solutions that are more tailored, effective, and centered around patient needs.

## Introduction and background

Exploring the dimensions: an introduction to 4D printing technology

4D printing denotes an additive manufacturing modality characterized by the fabrication of objects utilizing materials endowed with the capacity for self-metamorphosis or morphological adjustment in reaction to external stimuli during temporal progression. These stimuli encompass a spectrum of environmental variables, such as temperature, luminosity, moisture, and additional ambient factors [1]. The designation "4D" connotes the standard triad of spatial dimensions-length, width, and height-augmented by the temporal dimension. This temporal component facilitates the programmed transformation of printed artifacts, enabling predetermined alterations in shape or structural configuration post-fabrication. The prospective applications of this technology span diverse sectors, including aerospace, healthcare, architectural design, and consumer goods, indicating its substantial promise and versatility [2].

Evolution and milestones of 4D printing technology

Additive manufacturing has transformed the landscape of the manufacturing industry, offering the capability to produce intricate geometries with unprecedented ease. The emergence of 4D printing technology represents a novel advancement beyond traditional 3D printing, introducing the temporal dimension. This innovative extension enables materials to autonomously self-assemble, alter shape, or undergo transformation into new configurations over time. In circa 2013, Skylar Tibbits, an innovative scholar affiliated with the Massachusetts Institute of Technology (MIT), introduced the groundbreaking notion of "4D printing," envisioning a departure from the constraints of traditional 3D printing. Tibbits conceived of a manufacturing approach that integrates materials endowed with the unique ability for self-assembly or dynamic shape alteration over time, marking a pivotal moment in fabrication technology. The early part of the 2010s saw a surge in global exploration of 4D printing potentialities by various research entities, with MIT's Self-Assembly Lab, led by Tibbits, at the forefront of this movement [3]. Through the demonstration of proof-of-concept prototypes featuring self-folding structures and materials responsive to external stimuli, MIT's efforts showcased the transformative capabilities of 4D printing [4]. Despite challenges such as scalability and precise control over shape modulation, the extraordinary scientific potential of 4D printing suggests promising applications in fields like biomedical, medical, and dental sciences.

## Review

Beyond three dimensions: the rise of the fourth dimension of printing technology

**Figure 1:**
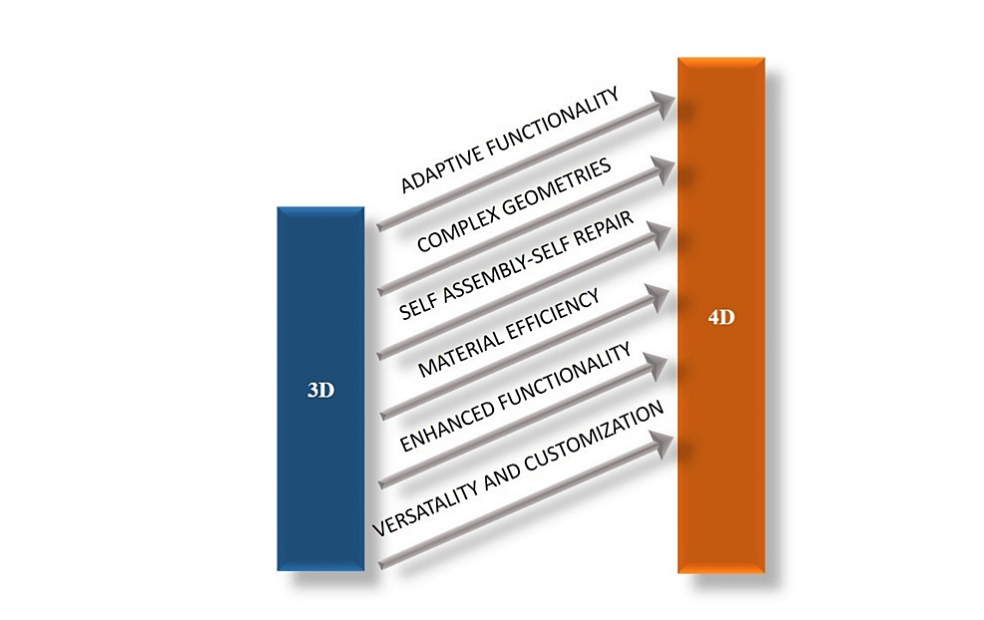
A depiction of the distinguishing features that differentiate 4D printing technology from 3D printing technology Credit: Image created by the author

4D printing presents numerous advantages over conventional 3D printing by integrating the temporal dimension into the manufacturing process and employing dynamic materials capable of undergoing shape alterations or transformations in reaction to external stimuli [5]. An elaborate discourse delineating the ways in which 4D printing outperforms 3D printing across several domains is mentioned in Figure 1.

Distinguishing Features

Adaptive functionality: A key benefit of 4D printing lies in its capacity to fabricate objects endowed with adaptive functionality. In contrast to the static nature of objects manufactured through 3D printing, those created through 4D printing exhibit the capability to undergo changes in shape, properties, or functionality over time, prompted by external stimuli such as fluctuations in temperature, exposure to light, variations in humidity levels, or other environmental influences. This inherent adaptability renders 4D-printed objects well-suited for diverse applications wherein dynamic responses are imperative [6]. For instance, dental implants fabricated through 4D printing can autonomously conform to temperature fluctuations, ensuring a consistently accurate and comfortable fit amidst changing environmental factors within the oral cavity. Furthermore, adaptive 4D-printed orthodontic appliances can incrementally alter alignment in alignment with patient progress, thereby enhancing treatment efficacy and precision beyond what static 3D-printed counterparts can achieve [7].

Complex geometries: Although 3D printing facilitates the production of intricate geometries with considerable convenience, 4D printing elevates this capability by permitting the generation of objects with heightened intricacy and dynamicity. Through leveraging the shape-altering attributes of dynamic materials, 4D printing can yield objects boasting intricate internal architectures or mobile components that would present challenges or feasibility issues when employing conventional manufacturing techniques. As an illustration, dental models fabricated via 4D printing can integrate responsive attributes, enabling the development of deeply individualized prosthetic solutions. Furthermore, the intrinsic pliability of 4D-printed substrates permits the fabrication of intricate configurations, including bespoke implants tailored to accommodate shifting oral dynamics, thus guaranteeing superior performance and durability [8].

Self-assembly and self-repair: 4D printing facilitates the production of objects capable of self-assembling or self-repairing devoid of external intervention. For instance, structures created through 4D printing could autonomously fold or unfold into predefined configurations or mend damage incurred from wear and tear over time. This innate ability for self-assembly and self-repair holds considerable ramifications for domains including robotics, infrastructure upkeep, and healthcare [9]. For instance, dental implants crafted through 4D printing may incorporate self-healing materials, enabling automated repair of minor impairments. This feature not only extends the longevity of the implants and diminishes the necessity for frequent interventions but also elevates patient comfort while mitigating the potential hazards associated with compromised prostheses. This attribute underscores a distinct advantage over conventional 3D-printed counterparts.

Material efficiency: A further benefit of 4D printing lies in its capacity for enhancing material efficiency. By harnessing the programmable shape-altering properties of dynamic materials, 4D printing can optimize the utilization of materials and diminish waste throughout the fabrication process. This attribute proves particularly advantageous in contexts where lightweight and resource-efficient structures are sought after, such as within the aerospace or automotive sectors. As an example, dental structures produced through 4D printing exhibit the capability to adaptively alter their shape and characteristics, consequently diminishing the requirement for surplus material consumption in the manufacturing process [10].

Enhanced functionality: 4D printing presents an opportunity to augment the functionality of printed objects beyond the capabilities achievable through 3D printing alone. Through the integration of sensors, actuators, or other functional elements into dynamic materials, 4D-printed objects can demonstrate advanced functionalities such as sensing, actuation, or communication. This broadens the horizon for the development of intelligent and interactive objects for diverse applications, encompassing realms such as healthcare, consumer electronics, and wearable technology [11].

Versatility and customization: 4D printing offers enhanced versatility and customization capabilities in contrast to 3D printing. By enabling precise control over the timing and extent of shape changes or transformations, 4D printing facilitates the production of tailor-made objects tailored to precise requirements or preferences. This inherent versatility and customization potential render 4D printing well-suited for a diverse array of applications spanning various industries, ranging from personalized medical implants to customizable consumer goods. For instance, dental appliances fabricated through 4D printing possess the capability to adaptively modify their characteristics in response to individual patient requirements and evolving oral circumstances, facilitating the creation of meticulously customized solutions [12].

General principles of 4D printing technology

**Figure 2:**
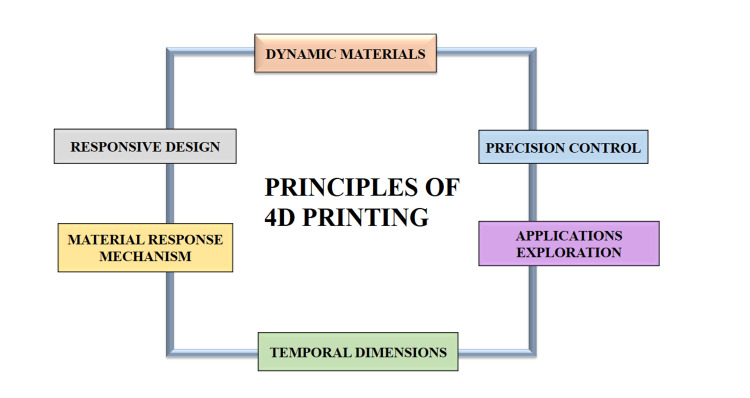
A depiction of the general principles of 4D printing technology Credit: Image created by the author

4D printing technology constitutes a notable progression beyond conventional 3D printing methodologies, incorporating the temporal aspect into the manufacturing process. Fundamentally, the principles underpinning 4D printing hinge upon the utilization of dynamic materials capable of undergoing alterations in shape or form in reaction to external stimuli [13]. An intricate elucidation of the underlying principles guiding 4D printing is mentioned in Figure 2.

Principles

Dynamic materials: Central to 4D printing is the utilization of dynamic materials endowed with the capability to undergo alterations in shape, properties, or functionality in response to particular external stimuli. Such materials encompass a variety of options, including shape-memory polymers, hydrogels, smart alloys, and other responsive substances [14,15].

Responsive design: The design process in 4D printing is meticulously crafted to capitalize on the distinctive characteristics of dynamic materials. Objects are meticulously engineered with predetermined shape-altering functionalities that are triggered by external stimuli such as fluctuations in temperature, exposure to light, variations in moisture levels, or magnetic fields.

Material response mechanism: Various dynamic materials demonstrate diverse response mechanisms to external stimuli. For instance, shape-memory polymers can undergo reversible alterations in shape upon heating or cooling, whereas hydrogels may expand or contract in reaction to fluctuations in humidity levels. Profound comprehension and effective utilization of these material response mechanisms are imperative in the realm of 4D printing [16].

Temporal dimensions: In contrast to conventional 3D printing, which yields static objects, 4D printing integrates the temporal dimension into the fabrication process. Printed objects are meticulously designed to undergo pre-defined shape alterations or transformations over time subsequent to fabrication, thereby incorporating the fourth dimension, time, into the process, hence the appellation "4D" (comprising three spatial dimensions alongside time) [17].

Precision control: Attaining meticulous control over the timing and extent of shape alterations or transformations holds paramount importance in the realm of 4D printing. This necessitates thorough deliberation of factors encompassing material characteristics, stimulus triggers, the geometry of the printed object, and environmental parameters to guarantee the attainment of the intended functionality [18].

Applications exploration: The potential applications of 4D printing are extensive and varied. For instance, in healthcare, implants fabricated through 4D printing could dynamically alter their shape to conform to precise anatomical structures. Ongoing research and development endeavors are continuously exploring and unveiling novel opportunities for this burgeoning technology across diverse industries [19].

**Figure 3:**
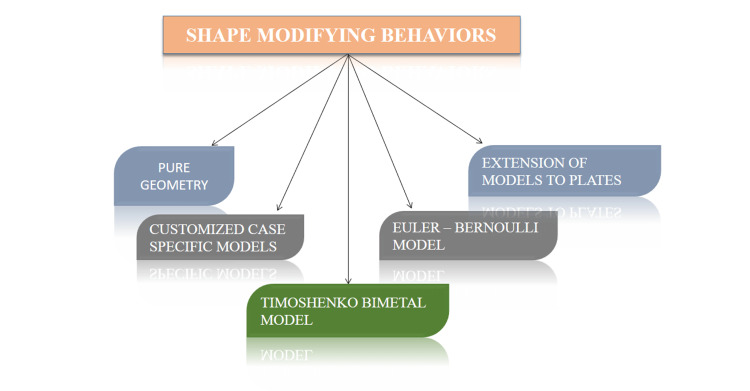
A depiction of the shape-modifying behaviors Credit: Image created by the author

The equations utilized or created in the realm of 4D printing to model the shape-morphing behaviors can be categorized into five distinct groups which are mentioned in Figure 3.

Laws of 4D printing technology

**Figure 4:**
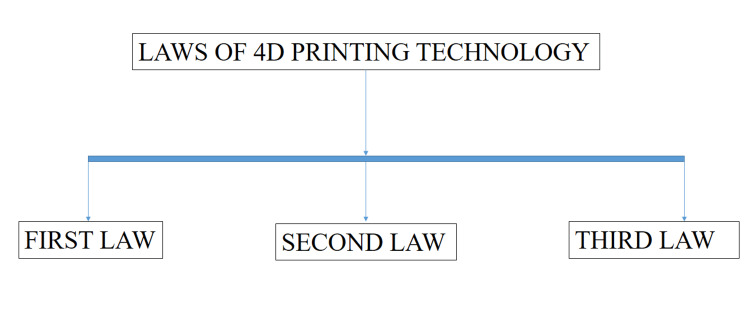
A depiction of the laws of 4D printing technology Credit: Image created by the author

The prognostication of the time-evolving characteristics, which constitute the fourth dimension, in any structure produced through 4D printing is imperative. Through an exhaustive and methodical exploration of 4D printing and its associated domains, we are able to discern three overarching principles that dictate the shape-altering tendencies of nearly all 4D structures, notwithstanding the diversity of materials and stimuli involved. These principles serve a dual purpose: first, to gain comprehension, and second, to formulate models and projections regarding the fourth dimension [20]. The laws of 4D printing technology are mentioned in Figure 4.

First Law

The vast array of shape-altering tendencies are observed in multi-material 4D-printed structures, including those influenced by thermo-responsive stimuli, electrochemical and thermal, ultrasound, enzymes, hydro, photothermal, and photochemical solvents. These factors can be attributed to a fundamental phenomenon known as relative expansion between active and passive materials. This relative expansion serves as the underlying mechanism for the intricate shape-morphing behaviors exhibited in 4D printing, such as twisting, coiling, and curling. These behaviors are made possible through the incorporation of various forms of anisotropy between active and passive materials and the fabrication of diverse heterogeneous structures.

Second Law

The shape-altering characteristics exhibited by the majority of multi-material 4D-printed structures are governed by four distinct physical mechanisms which include mass diffusion, thermal expansion, molecular transformation, and organic growth. These mechanisms are thoroughly examined, quantified, and integrated into the subsequent sections. Each of these mechanisms contributes to the relative expansion between active and passive materials, resulting in the ensuing shape-morphing behaviors when subjected to stimuli. While these stimuli typically originate externally, they can also arise internally.

Third Law

The time-evolving shape-altering characteristics observed in the majority of multi-material 4D-printed structures are regulated by two distinct "categories" of time constants. In the simplest scenario, where a multi-material 4D-printed structure comprises one active and one passive layer, these constants play a pivotal role.

**Figure 5:**
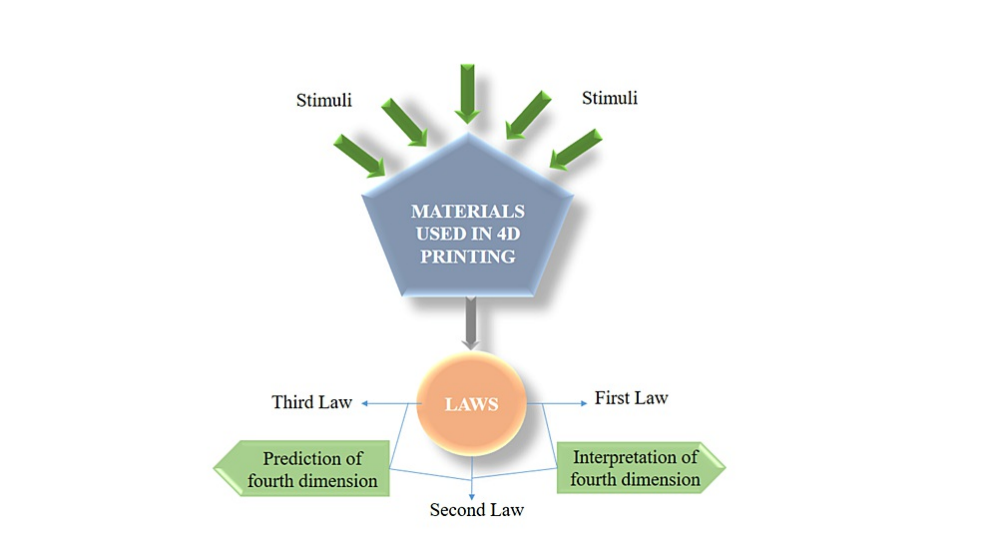
A depiction of the representation of 4D printing technology Credit: Image created by the author

The pictorial representation of 4D printing technology is mentioned in Figure 5.

Material considerations for 4D printing technology and its application in the field of dentistry

Material selection in 4D printing entails a meticulous evaluation of various factors to ensure the chosen material effectively accomplishes the desired shape-changing behavior. The process involves several key steps which include identifying the specific shape transformations required for the application and selecting a material that can respond appropriately to stimuli like heat, light, or moisture. Pinpointing the external stimulus that will trigger the shape change and ensuring the material is responsive under controlled conditions. Considering the mechanical, thermal, chemical, and biocompatibility properties of the material to ensure alignment with application requirements. Evaluating compatibility with the 3D printing process, including factors like printability and post-processing needs. Assessing cost-effectiveness and availability, while also conducting experimental validation to confirm the material's performance. Accounting for regulatory and safety considerations to ensure compliance with relevant standards and guidelines [21].

Shape Memory Polymers

Shape memory polymers (SMPs) represent a category of intelligent materials endowed with the capacity to revert from a deformed state to their original, predetermined form under specific external stimuli, such as changes in temperature, light exposure, or alterations in pH levels [22]. This capability of shape recovery arises from the temporary fixation of the polymer's macromolecular chains into an interim configuration, which can be prompted to return to its permanent state upon activation [23]. SMPs demonstrate attributes akin to both elasticity and plasticity, enabling them to undergo reversible deformation and recuperation multiple times without enduring lasting damage. These materials find diverse applications across sectors such as medicine, aerospace, textiles, and robotics, where their shape-altering characteristics are harnessed for purposes like actuation, sensing, and the creation of adaptable structures [24]. The utilization of SMPs in the field of dentistry is mentioned in Table 1.

**Table 1:** An illustration of the utilization of shape memory polymers in the field of dentistry PCL: Polycaprolactone; SMP: Smart memory polymers; PEG: Polyethylene glycol

Shape memory polymer	Inference	Applications in dentistry
Polycaprolactone (PCL)	Biodegradable polyester that exhibits shape memory behavior [25].	Dental implants, temporary crowns, and orthodontic devices can conform to the patient's anatomy and then revert to their original shape once implanted.
Polyurethane-based SMPs	Polyurethane-based shape memory polymers offer flexibility and biocompatibility, making them suitable for various dental applications.	Dental splints, aligners, and prosthetic devices that can adjust to the patient's mouth shape over time.
Methacrylate-based SMPs	Methacrylate-based shape memory polymers are commonly used in dental materials due to their excellent mechanical properties and biocompatibility.	Temporary bridges, denture bases, and orthodontic brackets.
Polyethylene glycol (PEG) based SMPs	PEG-based shape memory polymers are hydrophilic and can swell in the presence of water, making them suitable for dental applications where moisture responsiveness is required.	Dental adhesives, drug delivery systems, and oral tissue scaffolds that can adapt to the oral environment.

Hydrogels

Hydrogels employed in 4D printing represent interconnected networks of hydrophilic polymers with the ability to absorb and retain substantial quantities of water or biological fluids while preserving their structural integrity. These materials possess an inherent affinity for water, enabling them to expand when immersed in aqueous environments and undergo notable changes in volume in response to various environmental cues, including alterations in pH, temperature, light exposure, or electrical signals. Within the realm of 4D printing, hydrogels serve as essential components, functioning as both printing inks and supportive substrates to produce objects endowed with dynamic shape-shifting capabilities [26]. Through the integration of hydrogels into the printing process, intricate structures can be crafted that exhibit controlled deformation, bending, folding, or swelling over time. Such advancements hold promise for a myriad of applications across disciplines such as biomedicine, soft robotics, and tissue engineering [27]. The utilization of hydrogels in the field of dentistry is mentioned in Table 2.

**Table 2:** An illustration of the utilization of hydrogels in the field of dentistry PEG: Polyethylene glycol; GelMA: Gelatin methacrylate

Hydrogels	Inference	Applications in dentistry
Alginate hydrogels	Biocompatible, easy to handle.	Dental impressions and temporary restorations of custom-fitted dental devices and implants
Polyethylene glycol (PEG) hydrogels	Commonly used in tissue engineering and drug delivery due to their biocompatibility and tunable properties.	Dental scaffolds, oral drug delivery systems, or biocompatible coatings for dental implants.
Methacrylate-based hydrogels	Excellent mechanical properties and compatibility with dental restorative materials.	Fabrication of dental prostheses, orthodontic appliances, and oral tissue scaffolds.
Chitosan hydrogels	Possess antimicrobial properties and biocompatibility, making them suitable for dental applications such as wound healing and tissue regeneration.	Fabrication of dental implants, periodontal membranes, or bioactive dental materials.
Gelatin methacrylate (GelMA) hydrogels	Derived from gelatin and has been extensively studied for tissue engineering and regenerative medicine applications.	Dental scaffolds, gingival tissue substitutes, or bioprinted constructs for tooth regeneration.

Smart Elastomers

Smart elastomers utilized in 4D printing encompass a category of materials that amalgamate the characteristics of elastomers with sensitivity to external triggers. This amalgamation enables them to undergo deliberate alterations in shape over time. These materials demonstrate elastic properties, permitting reversible deformation when subjected to mechanical forces, while also possessing the capability to react to particular stimuli such as changes in temperature, light exposure, or pH levels. Within the realm of 4D printing, smart elastomers serve as key components in generating objects endowed with dynamic shape-shifting abilities [28]. During the printing process, the desired shape changes are encoded into the material's structure. Through the integration of smart elastomers into 4D printing, intricate structures can be crafted with the ability to adapt, deform, or reconfigure in response to environmental cues. This advancement holds promise for a wide array of applications in fields such as soft robotics, wearable technology, and biomedical implants [29]. The utilization of smart elastomers in the field of dentistry is mentioned in Table 3.

**Table 3:** An illustration of the utilization of smart elastomers in the field of dentistry UV: Ultraviolet

Smart elastomers	Inference	Applications in dentistry
Thermoresponsive elastomers	Change their shape or properties in response to temperature variations.	Dental devices that adapt to the oral environment, such as orthodontic aligners or customized mouthguards.
Light-responsive elastomers	Elastomers that respond to light stimuli, such as UV light, can be utilized in 4D printing for dental applications.	Light-responsive elastomers enable precise control over shape changes during printing and can be used to fabricate dental prostheses, temporary crowns, or oral drug delivery systems.
pH-sensitive elastomers	Elastomers that exhibit changes in shape or properties in response to variations in pH levels.	Oral drug delivery or tissue engineering. pH-sensitive elastomers enable targeted release of therapeutic agents or modulation of cellular responses within the oral cavity.
Stimuli-responsive hybrid elastomers	Hybrid elastomers that combine multiple responsiveness mechanisms, such as temperature and pH sensitivity, offer enhanced control over shape changes in 4D printing.	Tailored for specific dental applications, including personalized orthodontic devices, periodontal membranes, or dental adhesives.

Composites

Composites employed in 4D printing technology involve the integration of multiple components with diverse properties to craft structures capable of dynamic shape alterations over time. These composite materials typically comprise a base material fortified with embedded elements like fibers, particles, or additives, which confer specific functionalities or response capabilities [30]. In the realm of 4D printing, these composites are applied to manufacture objects endowed with shape transformations that can be programmed strategically by incorporating materials exhibiting varied reactions to external stimuli, such as temperature fluctuations, light exposure, moisture changes, or mechanical forces. Through leveraging the synergistic attributes of their constituent elements, composites facilitate the production of intricate structures capable of adapting, deforming, or reconfiguring in response to environmental stimuli. This attribute renders them versatile and functional across a broad spectrum of applications spanning aerospace, engineering, biomedical, and consumer product domains [31]. The utilization of composites in the field of dentistry is mentioned in Table 4.

**Table 4:** An illustration of the utilization of composites in the field of dentistry PLA: Polylactic acid; PGA: Polyglycolic acid

Composites	Inference	Applications in dentistry
Bioactive composites	incorporate bioactive materials, such as calcium phosphates or bioactive glasses, into a polymer matrix.	Bioactive composites promote remineralization and integration with natural tooth structures, making them suitable for dental restorations, fillings, or implants [32].
Antimicrobial composites	Composites containing antimicrobial agents, such as silver nanoparticles or quaternary ammonium compounds, help prevent bacterial colonization and reduce the risk of dental infections.	Dental prosthetics, orthodontic appliances, or periodontal membranes [33].
Biodegradable composites	composed of polymers such as polylactic acid (PLA) or polyglycolic acid (PGA) degrade over time and are absorbed by the body, making them suitable for temporary dental devices or drug delivery systems	Dental splints, sutures, and bone graft substitutes [34].
Hybrid composites	combine different types of materials, such as ceramics, metals, or polymers, to achieve specific mechanical, aesthetic, or biocompatible properties [35,36].	Dental crowns, bridges, or veneers that require enhanced strength and durability.
Thermoresponsive composites	containing thermoresponsive polymers undergo reversible shape changes in response to temperature variations.	Thermoresponsive composites are employed in 4D printing for dental appliances that adapt to oral temperature changes, such as denture bases or orthodontic aligners.

Conductive Materials

Conductive materials utilized in 4D printing technology are substances endowed with the capacity to conduct electricity. These materials are integrated into printed objects to confer electrical conductivity or responsiveness to external electrical stimuli. Typically, these materials comprise conductive particles such as graphene, carbon nanotubes, or metal nanoparticles dispersed within a polymer matrix or ink. In the realm of 4D printing, conductive materials serve to fashion objects with functional electrical properties, including sensors, actuators, or electronic circuits, which can be activated or regulated via electrical signals [37]. By integrating conductive materials into the printing process, it becomes feasible to manufacture intricate structures capable of detecting, transmitting, or modulating electrical signals, thus offering potential applications in fields such as electronics, wearable technology, and smart devices. The utilization of conductive materials in the field of dentistry is mentioned in Table 5.

**Table 5:** An illustration of the utilization of conductive materials in the field of dentistry CNT: Carbon nanotubes

Conductive materials	Inference	Applications in dentistry
Graphene	A single layer of carbon atoms arranged in a hexagonal lattice is known for its excellent electrical conductivity.	In dentistry, graphene-based conductive inks can be used to create dental sensors for monitoring parameters such as temperature, pH levels, or bacterial presence in the oral cavity [38].
Carbon nanotubes (CNTs)	Carbon nanotubes exhibit exceptional electrical conductivity and mechanical strength.	CNTs can be incorporated into dental materials to enhance their electrical properties or used to fabricate conductive pathways for electronic devices embedded within dental prostheses or orthodontic appliances [39].
Silver nanoparticles	Possess high electrical conductivity and antimicrobial properties.	Conductive dental adhesives, fillings, or coatings for dental implants with antibacterial functionalities.
polyaniline or polypyrrole	Electrical conductivity and flexibility, make them suitable for flexible electronic devices in dentistry.	Fabrication of flexible sensors or electrodes for intraoral diagnostic applications [40].
Metal nanoparticles	Metals such as gold or copper nanoparticles, exhibit good electrical conductivity and biocompatibility.	Metal nanoparticle-based inks can be used to create conductive patterns for electronic circuits or implantable sensors for monitoring dental health parameters.

Responsive Inks

Responsive inks utilized in 4D printing technology are specialized printing materials with dynamic characteristics that react to external stimuli. These inks are engineered to undergo specific alterations, such as changes in color, shape, conductivity, or mechanical attributes, in response to environmental cues like fluctuations in temperature, exposure to light, variations in humidity, or chemical interactions [41]. Typically composed of functional components such as intelligent polymers, nanoparticles, or reactive dyes dispersed within a carrier medium such as a polymer solution or solvent, responsive inks enable the production of objects with programmable behaviors that evolve over time. In the realm of 4D printing, these inks facilitate the fabrication of intricate structures whose responsive properties are predetermined within the ink formulation and activated by external triggers during the printing process [42]. By leveraging the capabilities of responsive inks, 4D printing technology enables the creation of complex structures capable of adapting, evolving, or self-assembling in response to changing environmental conditions, opening new avenues for innovation in fields including materials science, biomedicine, and consumer electronics. The utilization of responsive inks in the field of dentistry is mentioned in Table 6.

**Table 6:** An illustration of the utilization of responsive inks in the field of dentistry

Responsive inks	Inference	Applications in dentistry
Thermochromic inks	These inks change color in response to temperature variations, allowing for the creation of dental devices that indicate temperature changes in the oral cavity.	It can be incorporated into dental appliances like mouthguards or orthodontic aligners to alert users to potential issues such as temperature-sensitive dental conditions or improper fit.
Hydrochromic inks	Hydrochromic inks alter their color or transparency when exposed to moisture, making them suitable for dental applications where moisture levels play a role, such as detecting saliva or plaque accumulation.	Fabrication of dental devices that provide visual feedback on oral hygiene or moisture levels in the mouth.
pH-sensitive inks	Change color in response to variations in pH levels, allowing for the creation of dental devices that monitor oral pH balance.	These inks can be integrated into dental materials such as dental adhesives, restorations, or remineralization agents to indicate changes in oral acidity or alkalinity, aiding in the prevention of dental caries or enamel erosion [43].
Antimicrobial inks	Antimicrobial inks contain agents that inhibit bacterial growth or promote oral health, making them suitable for dental applications where bacterial colonization is a concern.	Fabrication of dental devices with built-in antimicrobial properties, such as dental implants, prosthetics, or orthodontic appliances, to prevent infections or promote healing in the oral cavity.
Smart polymer inks	They undergo reversible changes in shape, stiffness, or conductivity in response to external stimuli such as temperature, light, or moisture.	Fabrication of dental devices with dynamic properties, such as shape-changing orthodontic appliances or self-adjusting dental materials that adapt to changes in the oral environment [44].

Biodegradable Polymers

Biodegradable polymers employed in 4D printing technology are organic-based materials engineered to naturally decompose into harmless substances when exposed to environmental factors like moisture, heat, or microbial activity. These polymers are specially designed to undergo regulated degradation, resulting in the gradual breakdown of printed items into environmentally safe components [45]. In 4D printing, these polymers serve as printing substrates to create objects with shape-changing capabilities over time, where the degradation process influences the objects' temporal behaviors. By integrating biodegradable polymers into 4D printing processes, it becomes feasible to produce eco-friendly structures capable of adjusting, evolving, or disintegrating over time. Such advancements hold promise for applications in various sectors, including biomedical implants, environmental monitoring devices, and disposable consumer goods. The utilization of biodegradable polymers in the field of dentistry is mentioned in Table 7.

**Table 7:** An illustration of the utilization of biodegradable polymers in the field of dentistry PLA: Polylactic acid; PGA: Polyglycolic acid; PCL: Polycaprolactone; PHA: Polyhydroxyalkanoates

Biodegradable polymers	Inference	Applications in dentistry
Polylactic acid (PLA)	Biodegradable polymer derived from renewable resources such as corn starch or sugarcane. It is widely used in 4D printing for dental applications due to its biocompatibility, mechanical strength, and ability to degrade into non-toxic lactic acid under natural environmental conditions [46].	Fabrication of dental implants, temporary crowns, or orthodontic devices [47,48].
Polyglycolic acid (PGA)	Biodegradable polymers are commonly used in tissue engineering and drug delivery applications.	To create biodegradable scaffolds for periodontal tissue regeneration or controlled-release systems for dental therapeutics [49].
Polycaprolactone (PCL)	Biodegradable polyester with a slow degradation rate, making it suitable for long-term dental applications.	To fabricate dental splints, bone graft substitutes, or customized dental implants that gradually degrade and integrate with surrounding tissues over time [50].
Polyhydroxyalkanoates (PHA)	A group of biodegradable polymers produced by bacterial fermentation of renewable feedstocks. They exhibit biocompatibility and versatility in 4D printing for dental applications.	Fabrication of biodegradable sutures, drug delivery systems, or tissue scaffolds for oral tissue regeneration.
Chitosan	Biodegradable polysaccharide derived from chitin, a natural polymer found in the exoskeleton of crustaceans.	Create biodegradable films, membranes, or drug-delivery vehicles for periodontal therapy or oral wound healing [51].

Advantages of 4D printing technology

4D printing technology, with its unique capability to fabricate objects capable of altering shape or functionality in response to external stimuli, presents numerous distinct advantages within the domain of dentistry.

Customization

A paramount benefit of integrating 4D printing into dentistry lies in its unparalleled capacity for customization. This technology empowers dental practitioners to precisely tailor structures and appliances to harmonize with the singular anatomy and requirements of individual patients, thereby enhancing fit, comfort, and functionality.

Intricate Geometries

4D printing facilitates the production of dental devices featuring intricate geometries that would pose considerable challenges or be unattainable through conventional manufacturing methods. Such capabilities enable the creation of highly intricate dental implants, prosthetics, and orthodontic appliances, elevating the sophistication of treatment options.

Patient-Centric Solutions

By harnessing 4D printing, dental professionals can devise treatment solutions meticulously tailored to the precise needs and oral conditions of each patient. This personalized approach not only augments treatment efficacy and patient satisfaction but also mitigates the likelihood of complications arising during dental interventions.

Material Efficiency

In contrast to traditional subtractive manufacturing approaches notorious for generating substantial material waste, 4D printing operates on an additive principle, utilizing only the requisite amount of material. Consequently, this methodological shift contributes to diminished material waste and reduced environmental impact in dental manufacturing processes [52].

Enhanced Efficiency and Time Management

Despite inherent processing time constraints, the integration of 4D printing into dental workflows has the potential to streamline operations by consolidating multiple manufacturing stages into a cohesive process. This heightened efficiency translates into time savings throughout the fabrication and delivery of dental devices, benefiting both patients and dental practitioners.

Cutting-Edge Materials

Progressions in 4D printing technology have spurred the development of novel materials tailored specifically for dental applications. These advanced materials boast superior biocompatibility, durability, and flexibility, thereby augmenting the performance and longevity of dental devices.

Minimally Invasive Techniques

4D-printed dental devices facilitate minimally invasive treatment modalities by conforming precisely to the patient's anatomical contours, thereby minimizing the necessity for extensive surgical interventions. Consequently, patients experience reduced trauma, accelerated recovery periods, and overall enhanced treatment experiences.

Research and Development Opportunities

The versatility inherent in 4D printing technology serves as a catalyst for innovation within dental research and development endeavors. Researchers are afforded the opportunity to explore avant-garde designs, materials, and functionalities, thereby addressing unmet clinical needs and propelling the evolution of dental science.

Collectively, the advantages conferred by 4D printing in dentistry encapsulate a spectrum of benefits including personalized customization, geometric intricacy, operational efficiency, environmental sustainability, and pioneering innovation, all of which converge to elevate patient care standards and treatment outcomes within the dental sphere.

Limitations of 4D printing technology

4D printing technology, which entails the fabrication of objects capable of morphing in shape or behavior over time in response to external stimuli, holds considerable promise across diverse fields, including dentistry. Nonetheless, akin to any nascent technological innovation, it is not devoid of limitations, particularly within the realm of dental applications. Several constraints specific to 4D printing in dentistry are discernible.

Material Selection

The materials utilized in 4D printing must conform to stringent criteria regarding biocompatibility, durability, and flexibility to be deemed suitable for dental purposes. The restricted availability of such materials may impede the advancement of intricate dental structures.

Resolution and Accuracy

Attaining optimal resolution and precision in 4D printing processes assumes paramount importance in crafting exacting dental implants, prosthetics, or orthodontic apparatuses. Existing constraints in printing resolution could potentially compromise the fit, functionality, and enduring efficacy of dental constructs.

Processing Time

The procedural duration encompassing both printing and activation phases in 4D printing can be notably protracted. Dental establishments necessitate streamlined workflows to ensure the expeditious delivery of bespoke dental solutions, a task rendered challenging by prolonged fabrication durations.

Cost

4D printing technology remains relatively costly compared to conventional manufacturing methodologies prevalent in dentistry. The elevated expenses associated with equipment procurement and material acquisition might curtail widespread adoption, particularly among smaller dental practices or in developing regions.

Complexity of Design and Fabrication

The intricate geometries and multifaceted functionality inherent in designing and fabricating 4D-printed dental devices mandate specialized expertise and knowledge. Dentists and dental technicians may necessitate supplementary training to effectively harness the full potential of this technology [1].

Regulatory Approval and Standardization

Negotiating the labyrinthine regulatory approval processes pertinent to 4D-printed dental products can prove intricate and time-intensive. Establishing standardized protocols governing manufacturing, quality control, and safety assessment assumes paramount importance in ensuring compliance with regulatory requisites [53].

Long-Term Performance

A comprehensive evaluation of the enduring performance and biocompatibility of 4D-printed dental devices is imperative. Delving into the mechanical properties, degradation kinetics, and tissue response of 4D-printed materials is indispensable for appraising their suitability for prolonged clinical utilization.

Notwithstanding these constraints, ongoing research endeavors and technological strides are progressively tackling many of these impediments, thereby augmenting the capabilities and applications of 4D printing in dentistry. Through further refinement and advancement, 4D printing stands poised to revolutionize diverse facets of dental care, proffering tailored and functional solutions to patients.

Future directions

The future potential of 4D printing in dentistry is vast, spanning multiple critical areas of advancement. This includes the transformation of dental prosthetics, where dynamic devices could adapt to oral changes, enhancing patient comfort and functionality [54]. Orthodontic treatments may become more efficient and personalized through customizable appliances adjusting to tooth alignment. Moreover, 4D printing's integration with biofabrication techniques offers possibilities for tissue regeneration, addressing conditions like periodontal disease. Additionally, 4D-printed dental devices could deliver localized therapeutic agents, improving treatment precision while minimizing systemic effects. Precision surgical guides and implant components could be tailored to individual anatomy, enhancing procedure accuracy and patient outcomes. In education, 4D printing enables immersive learning experiences, while advancements in digital dentistry could facilitate remote care delivery, expanding access to high-quality dental solutions. This trajectory of innovation underscores the transformative potential of 4D printing in dentistry, requiring continued research, collaboration, and technological refinement to realize its full impact.

## Conclusions

The emergence of 4D printing technology in dentistry stands poised to transform the landscape of dental healthcare delivery and patient experience. This cutting-edge technology presents a wide array of opportunities, ranging from the creation of dynamic dental prosthetics to tailored orthodontic interventions and novel approaches to tissue regeneration. Through its capacity to drive innovation, enhance treatment precision, and widen the availability of sophisticated dental solutions, 4D printing heralds a significant advancement in the field. Ongoing research, collaboration, and refinement of this technology will be crucial in fully unlocking its potential to reshape the future of dental care.
